# Role of Advanced Glycation End Products in Intervertebral Disc Degeneration: Mechanism and Therapeutic Potential

**DOI:** 10.1155/2022/7299005

**Published:** 2022-12-17

**Authors:** Fengguang Yang, Daxue Zhu, Zhaoheng Wang, Yingping Ma, Liangzeng Huang, Xuewen Kang, Bing Ma

**Affiliations:** ^1^Department of Orthopedics, Lanzhou University Second Hospital, Lanzhou, China; ^2^The Second Clinical Medical College, Lanzhou University, Lanzhou, China; ^3^Key Laboratory of Orthopedics Disease of Gansu Province, Lanzhou University Second Hospital, Lanzhou, China; ^4^The International Cooperation Base of Gansu Province for the Pain Research in Spinal Disorders, Lanzhou, China; ^5^Department of Orthopedics, Gansu Provincial Hospital, Lanzhou, Gansu 730000, China

## Abstract

The incidence of low back pain caused by lumbar disc degeneration is high, and it can lead to loss of work ability and impose heavy social and economic burdens. The pathogenesis of low back pain is unclear, and there are no effective treatments. With age, the deposition of advanced glycation end products (AGEs) in intervertebral disc (IVD) gradually increases and is accelerated by diabetes and a high-AGEs diet, leading to destruction of the annulus fibrosus (AF), nucleus pulposus (NP), and cartilage endplate (CEP) and finally intervertebral disc degeneration (IDD). Reducing the accumulation of AGEs in IVD and blocking the transmission of downstream signals caused by AGEs have a significant effect on alleviating IDD. In this review, we summarize the mechanism by which AGEs induce IDD and potential treatment strategies.

## 1. Introduction

Lumbar intervertebral disc degeneration is considered to be the main cause of low back pain [1]. Low back pain refers to the pain in the lumbar spine. If the spinal cord or nerve roots are compressed, discomfort such as pain can also affect the lower limbs. In severe cases, the patient's mobility may be impaired. Low back pain is currently the most common musculoskeletal disease, with approximately 80% of the world's population experiencing low back pain at some point in their lives [2, 3], and it is the main reason why adults see physicians [4]. It is estimated that more than 600 million patients worldwide have low back pain, imposing considerable social and economic burdens [4, 5]. It is thus important to clarify the mechanism of intervertebral disc degeneration (IDD) and develop effective treatments.

The intervertebral disc (IVD) is the fibrocartilage tissue connecting the vertebral bodies of the spine and is comprised of the annulus fibrosus (AF), nucleus pulposus (NP), and cartilage endplate (CEP). The IVD plays an important role in bearing the impact of body movement, absorbing shocks, and distributing mechanical loads along the spine [6, 7]. The AF is composed of a ring rich in type I collagen, which is wound in a highly orderly manner around the NP. The collagen fibers are arranged at alternating angles to resist the circumferential stress on the NP during bending and twisting of the body and prevent its lateral displacement and collapse [8, 9]. The NP is located in the center of the IVD and is comprised of about 80% water, as a result of the permeability gradient of proteoglycan. The NP also contains an arrangement frame composed of type II collagen and elastin fibers, which combines with proteoglycans and transmits compressive stress to the AF and CEP [8, 10]. The CEP is the separation interface between the IVD and adjacent vertebral bodies and is mainly composed of a transparent hyaline cartilage matrix similar to joint cartilage [11]. IVD is nonvascular tissue. The transport of nutrients and metabolic wastes depends on the osmotic capacity of the CEP. The cell density of the NP is low (~3000/mm^3^) [12, 13]. The IVD's lack of perfusion and low cell density make it vulnerable to damage, and it has limited potential to repair such damage, causing accumulation of metabolic waste [14].

IDD is defined as the accumulation of degenerative factors leading to inappropriate cellular responses, which aggravates disease development and leads to loss of biological structural support and function [15]. Factors such as age, smoking, infection, biomechanical abnormalities, and malnutrition are implicated in IDD [16, 17]. In IDD, proinflammatory factors secreted by NP and AF cells, macrophages, T cells, and neutrophils [18–20] trigger a series of pathogenic reactions of IVD cells, promoting autophagy, senescence, and apoptosis [17, 21, 22]. IDD is characterized by changes in biochemical components and the resulting loss of biomechanical properties [23, 24], leading to a series of spinal diseases (such as intervertebral disc herniation and spinal stenosis).

Advanced glycation end products (AGEs) are metabolic derivatives of nonenzymatic reactions that occur between reducing sugars, free amines (mainly protein *α*-NH2 or *ε*-NH2 groups), and amino groups (from lipids and nucleic acids) [25]. AGEs modified by proteins undergo structural changes due to charge changes and cross-linking formation. This affects enzyme activity and function [26]. Therefore, the irreversible formation of AGEs results in their accumulation, especially in the presence of longevity proteins (e.g., collagen, serum albumin, and lens crystal) [27], leading to tissue damage and degeneration. AGEs are implicated in diabetes [28], cardiovascular diseases [29], kidney diseases [30], neurodegenerative diseases [27], and some cancers [31]. Here, we review the role of AGEs in IDD, focusing on the molecular mechanisms and therapeutic potential.

## 2. Advanced Glycation End Products (AGEs)

**Figure 1:**
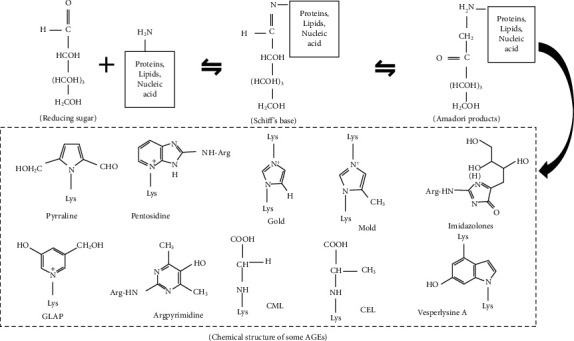
The general process of AGEs formation. CML: N-carboxymethyllysine; CEL: N-carboxyethyllysine; GOLD: glyoxal-lysine dimer; MOLD: methylglyoxal-lysine dimer; GLAP: glyceraldehyde-derived pyridinium compound.

AGEs are a group of heterogeneous compounds formed by the nonenzymatic reactions. They are formed by the nonenzymatic glycation of free amino groups of proteins, lipids, and nucleic acids, mainly via reducing sugars and reactive aldehydes [32, 33]. The intermediate steps in AGE formation involve a series of rearrangement and cyclization reactions [34]. The first is the nonenzymatic reaction of a reducing sugar and an amino group to produce an unstable Schiff base. This reaction is reversible until the equilibrium is reached. The unstable Schiff base undergoes rearrangement to form a more stable Amadori product [35]. The Amadori products undergo a series of reactions, rearrangements, and dehydrations to produce highly reactive dicarbonyl compounds, such as methylglyoxal (MG), glyoxal (GO), or deoxyglucone (1-deoxyglucone [1-DG] and 3-deoxyglucone [3-DG]) [35–37]. Carbonyl stress is caused by continuous accumulation of dicarbonyl compounds [38]. These dicarbonyl compounds undergo oxidation, dehydration, and cyclization reactions to form AGEs [27, 39, 40] (Figure 1).

AGEs may originate from exogenous sources, such as daily diet and smoking. A large number of AGEs are produced in food processing, particularly baking, frying, and barbecuing, and some are found in raw animal-derived food [41, 42]. Nɛ-Carboxymethyllysine (CML), pentosidine, methylglyoxal-lysine dimers (MOLD), and pyrrolidine are common AGEs in food [43]. AGEs are related to several age-related diseases [44]. Eating habits are an important variable, and only about 10% of ingested AGEs are absorbed and distributed in the tissues [45, 46]. More than 70% of AGEs escape absorption, because their cross-linking renders them resistant to enzymatic or acid hydrolysis [47]. Compared with nonsmokers, the serum AGEs levels of smokers are significantly higher [48], and AGEs formation from tobacco glycotoxins requires only a few hours [49].

AGEs are chemically modified proteins, lipids, or nucleic acids with stable chemical properties. AGEs may exert their effects as follows: (1) glycosylated proteins act as ligands to activate cell membrane receptors (such as RAGE), leading to oxidative stress and inflammation [50]; (2) glycosylated proteins form cross-links with other proteins, altering their activities and hardness [51]; and (3) saccharifying agents saccharify proteins, affecting their biological functions [32]. AGEs can also spread free radical reactions, thereby further damaging proteins, lipids, and/or nucleic acids [27].

More than 20 AGEs have been found in human blood, tissues, and food [52]. According to their chemical structure and fluorescence, they can be divided into four categories [52]: (1) fluorescent and cross-linked, (2) nonfluorescent and non-cross-linked, (3) nonfluorescent protein cross-linked, and (4) fluorescent non-cross-linked. The characteristics of several AGEs are listed in Table 1.

## 3. Effect of AGEs on IVD

### 3.1. Nucleus Pulposus Cells (NP Cells)

NP cells are the main functional cells of the NP. The young, healthy human IVD contains notochord cells, which originate from the vacuolar cells of the embryonic notochord, and NP cells. The latter are small spherical cells with a unique phenotype similar to articular chondrocytes but express specific markers (e.g., ovos2, CA12, CD24, HIF-1*α*, and cytokeratin 8/18/19) [53–55]. The cell density of the NP is low (~3000/mm^3^) [12, 13]. NP cells synthesize and secrete extracellular matrix (ECM) rich in proteoglycan, type II collagen, and hyaluronic acid (HA), which maintains the osmotic pressure, and so also the biomechanical properties, of the spine [56].

AGEs accumulation in the IVD increases with age [57]. AGEs reduce the viability of NP cells by a variety of mechanisms, affect their proliferation, and promote their apoptosis [58]. The accumulation of AGEs in IVD tissue affects endoplasmic reticulum (ER) homeostasis [59]. ER-phagy is a type of selective autophagy. Some ER fragments are phagocytized by autophagosomes via specific receptors and transported to lysosomes for degradation, to restore the cellular energy level and ER homeostasis [60]. AGEs can trigger the accumulation of reactive oxygen species (ROS) in NP cells, activating ER-phagy mediated by FAM134B (a mammalian ER-phagy receptor). The overexpression of FAM134B alleviates ROS accumulation, apoptosis, and senescence in AGEs-treated NP cells [59]. The ER is responsible for protein synthesis, maturation, and quality control. Genetic and environmental pressures affect protein folding in the ER, resulting in the accumulation of unfolded/misfolded proteins, i.e., ER stress. Continuous ER stress can trigger cell self-destruction [61, 62]. AGEs induced a persistent increase of cytosolic Ca^2+^ ([Ca^2+^]c) and depletion of ER cavity Ca^2+^ ([Ca^2+^]er) in NP cells in a concentration- and time-dependent manner, resulting in ER stress [62]. AGEs alter the activity of ER Ca^2+^ channels in NP cells, including 1,4,5-triphosphate receptor channels (IP3R) and ryanodine receptor channels (RyR), and ER Ca^2+^-reuptake pumps, such as sarco/endoplasmic reticulum Ca^2+^-ATPase (SERCA) [63–65]. Pharmacological blockade of ER Ca^2+^ release by Ca^2+^ antagonists can improve the Ca^2+^ imbalance, ER stress, and apoptosis in NP cells and reduce the progression of IDD *in vivo* [63]. In addition, mesenchymal stem cell-derived exosomes (MSC-exos) can prevent the apoptosis of NP cells induced by ER stress by activating the AKT and ERK signaling pathways [63]. AGEs induce ER stress and activate the unfolded protein response (UPR) via key transmembrane proteins in the ER [66, 67]. After initiation of the UPR, the downstream C/EBP homologous protein (CHOP) is transcriptionally activated and controls the expression of apoptosis-related genes, thus inducing apoptosis under severe ER stress [68]. MSC-exos regulate ER stress by modulating the UPR and CHOP expression [69].

Because it constitutes the largest avascular tissue, anaerobic glycolysis is considered the main pathway of energy metabolism in the IVD [14, 70]. The NP is a hypoxic tissue due to a lack of vascularization [71], so NP cells stably express hypoxia-inducible factor-1*α* (HIF-1*α*) [72]. HIF-1*α* interacts with transcriptional coactivators, including p300/CBP, to upregulate genes such as glyceraldehyde-3-phosphate dehydrogenase (GAPDH), glucose transporter (GLUT)-1, and GLUT-3, thus driving glycolytic metabolism [73–75]. HIF-1*α* expression is a biomarker of normal NP cells [72]. Treatment of NP cells with AGEs impaired the stability of HIF-1*α*. Following AGEs treatment, the receptor for activated C-kinase 1 (RACK1) competes with heat shock protein 90 (HSP90) for binding to HIF-1*α*, resulting in posttranslational HIF-1*α* degradation and RACK1-mediated proteasomal degradation, independently of the canonical iron-dependent prolyl-hydroxylase domain- (PHD-) mediated degradation pathway. Under normoxic conditions, PHD proteins promote HIF-1*α* degradation by the 26S proteasome [76, 77]. The degradation of HIF-1*α* disrupts the biological function of NP cells and promotes IDD.

The IVD generates energy by anaerobic sugar degradation, which has nothing to do with mitochondrial pathway, but mitochondria may be involved in the adaptive changes in the metabolic process of NP cells [78, 79]. The mitochondrial pathway regulates apoptosis via changes in mitochondrial membrane permeability and release of proapoptotic proteins [80]. AGEs increase the production of mitochondrial ROS, prolong the activation time of mitochondrial permeability transition pores, increase the level of mitochondrial Bax, decrease the Bcl-2 level, increase the intercellular ROS level, and promote the apoptosis of NP cells. This may involve functional impairment of SIRT3 (an NAD^+^-dependent deacetylase, which has deacetylase activity and maintains mitochondrial redox homeostasis and functional integrity) [58, 81, 82]. The above-mentioned changes in NP cells caused by AGEs can be rescued by nicotinamide mononucleotide (NMN) through the adenosine monophosphate-activated protein kinase (AMPK)/peroxisome proliferator-activated receptor-*γ* coactivator 1*α* (PGC-1*α*) pathway, which restores SIRT3 function [58]. AGEs treatment of NP cells leads to mitochondrial dysfunction, which may involve mitochondrial quality control pathways [83]; this warrants further investigation.

**Figure 2:**
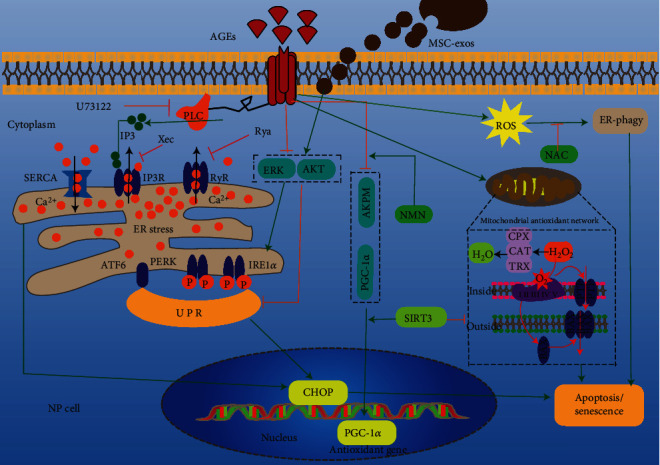
Mechanisms of AGEs-induced apoptosis (or senescence) of nucleus pulposus cells through endoplasmic reticulum and mitochondrial pathways. AGEs: advanced glycation end products; IP3: inositol 1,4,5-trisphosphate; PLC: phospholipase C; Rya: ryanodine; Xec: xestospongin C; RyR: ryanodine receptor; IP3R: inositol 1,4,5-triphosphate receptor (U73122, xec, and RYA are calcium antagonists of PLC, IP3R, and RyR, respectively); SERCA: sarco/endoplasmic reticulum Ca^2+^-ATPase; UPR: unfolded protein response; ROS: reactive oxygen species; CHOP: C/EBP homologous protein; ATF6: activating transcription factor 6; PERK: protein kinase-like endoplasmic reticulum kinase; IRE1*α*: inositol-requiring protein 1*α*; MSC-exos: mesenchymal stem cells-exosomes; AKT: protein kinase B; ERK: extracellular regulated protein kinases; AMPK: adenosine monophosphate-activated protein kinase; PGC-1*α*: peroxisome proliferator-activated receptor-*γ* coactivator 1*α*; NMN: nicotinamide mononucleotide; SIRT3: Sirtuin3; NAC: N-acetyl-L-cysteine; PTP: permeability transition pore; ER stress: endoplasmic reticulum stress; ER-phagy: endoplasmic reticulum-phagy.

In conclusion, ER stress/phagy and mitochondrial dysfunction are involved in AGEs-induced apoptosis of NP cells (Figure 2), and epigenetic modification may also be implicated [84, 85]. Therefore, several pathways are involved in AGEs-mediated damage to NP cells.

### 3.2. ECM Metabolism

Proteoglycan and type II collagen are the main components of the ECM of the IVD, and they maintain its osmotic pressure. Indeed, they are the material basis of the biomechanical properties of the IVD [56]. ECM catabolism and anabolism are in dynamic balance in the healthy IVD. However, in IDD, ECM catabolism is greater than anabolism [86]. Matrix metalloproteinases (MMPs) and a disintegrin and metalloproteinase with thrombospondin motifs (ADAMTS)—including MMP-2, MMP-3, MMP-9, MMP-13, ADAMTS-4, and ADAMTS-5—are the main catabolic enzymes in the NP [87, 88]. Decreased synthesis and increased catabolism of NP cells are important in ECM degradation. AGEs accumulation increases with age [57], triggering catabolism in IVD cells [89].

**Figure 3:**
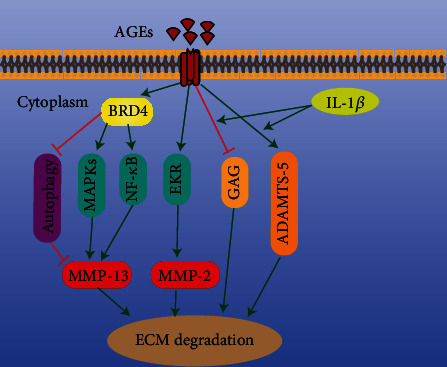
Mechanism of AGEs-induced ECM degradation. AGEs: advanced glycation end products; BRD4: bromodomain-containing protein 4; ERK: extracellular regulated protein kinase; MAPK: mitogen-activated protein kinase; NF-*κ*B: nuclear factor kappa-B; GAG: glycosaminoglycan; ADAMTS-5: a disintegrin and metalloproteinase with thrombospondin motif-5.

Immunohistochemistry confirmed the existence of AGEs and the receptor for advanced glycation end products (RAGE) in degenerating IVDs of human and oxtail. AGEs bind to RAGE and inhibit aggrecan secretion, which may be related to an inflammatory environment [89, 90]. The formation of endogenous AGEs occurs slowly during normal aging and is partly driven by sugar. In diabetes, the increase of blood sugar will accelerate the accumulation of AGEs [91]. The accumulation of AGEs in NP initiates the increase of MMP-2 expression related to ERK signaling pathway and promotes ECM decomposition [88]. Bromodomain-containing protein 4 (BRD4) passes MAPK and NF-*κ*B signals and activates autophagy by upregulating MMP-13 in the diabetic IVD. Inhibition of BRD4 prevented ECM degradation in diabetic rats [88]. ADAMTS-5 and MMP-13 were upregulated in diabetic mice, and anti-inflammatory (pentosan polysulfate) and AGEs inhibitors (pyridoxamine) were effective [92]. The glycosaminoglycan (GAG) content in the IVD of diabetes rats is decreased significantly, which is related to endplate sclerosis and AGEs [93]. However, its relationship with the expression of catabolic enzymes in the ECM is unclear. In the IVD, AGEs accumulate mainly in long-lived proteins (e.g., aggrecan and collagen), which are chemically modified to prevent their repair and renewal [57]. The result is upregulation of matrix catabolic enzymes, promotion of ECM degradation, and acceleration of IDD (Figure 3).

### 3.3. Annulus Fibrosus(AF)

**Figure 4:**
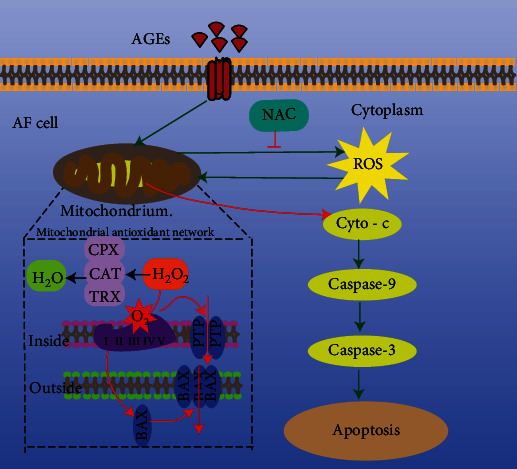
Mechanisms of AGEs-induced apoptosis of AF cells through mitochondrial pathways. AGEs: advanced glycation end products; NAC: N-acetyl-L-cysteine; ROS: reactive oxygen species; Cyto-c: cytochrome c.

The AF is composed of 15–25 0.14–0.52 mm thick layers of fiber bundles arranged in a cross [94]. The AF comprises 20% proteoglycan and 60% collagen [95], mainly type I collagen arranged as concentric rings. The AF can be divided into the external AF, mainly composed of type I collagen fibers with high tensile strength, and the internal AF, which is the transitional area between the external AF and NP and has low density and little tissue [96]. AF cells have the characteristics of mesenchymal-derived long fibroblasts [97]. AGEs inhibit the proliferation and induce the apoptosis of AF cells [98]. AGEs not only significantly upregulated proapoptotic Bax and downregulated antiapoptotic Bcl-2 in AF cells but also promoted the release of cytochrome c (Cyto-c) from mitochondria to cytoplasm. This results in activation of caspase-9 and caspase-3, increases the level of reactive oxygen species (ROS), and reduces the mitochondrial membrane potential. These effects are reversed by the antioxidant, N-acetyl-L-cysteine (NAC) [98]. Therefore, the mitochondrial pathway is involved in age-induced apoptosis of AF cells (Figure 4).

Collagen fibers in the AF are arranged at alternating angles to resist the circumferential stress of the NP and prevent its lateral displacement and collapse [8, 9]. Animal experiments showed that a high-AGEs diet can lead to marked accumulation of AGEs in the IVD. Two-photon imaging indicated that collagen damage in the fiber ring was increased by a high-AGEs diet, and the damage was significantly greater in females than in males [99]. Another study using a similar method showed that dietary AGEs increase destruction of collagen fibers and decrease the total collagen level in a RAGE-dependent manner, which was caused by catabolic processes other than cross-linking [100]. However, it is puzzling and interesting that the latter study is not gender dependent under basically similar research conditions. The researchers think that the possible causes are as follows: AGEs may involve age receptor interacting with estrogen [99]; the local effects of AGEs on AF collagen may be gender independent, while other spinal tissues and characteristics have age-related and sex-dependent effects [100]. Whether the effect of AGEs on AF collagen is gender dependent is controversial, warranting further research. In summary, AGEs accumulation in the IVD damages AF collagen, promotes collagen degradation, and is thus implicated in IDD.

### 3.4. Cartilage Endplates (CEP)

The IVD is connected to the adjacent vertebral body via the cartilage endplates (CEPs) on the upper and lower sides. The IVD is the largest avascular tissue, and its transportation of nutrients and metabolic waste depends on infiltration of the CEP [101–103]. Any factor that affects infiltration of the CEP may trigger IVD and ultimately IDD. The increase of blood glucose will accelerate the accumulation of AGEs [91]. In diabetic nonobese mice, micro-X-ray computed tomography showed that CEP thickness increased by 21% and its porosity decreased by 41%. The change of CEP microarchitecture was significantly correlated with the GAG content, and both oxidative stress and RAGE expression increased [93]. Activation of the AGEs/RAGE axis may play a role in AGEs-mediated CEP sclerosis. The level of oxidative stress is increased by AGEs-induced pathological changes in target sites (e.g., the NP, AF, and CEP), implicating the mitochondrial pathway in the pathogenic effects of AGEs.

Chronic (18 months of postweaning) nondiabetic mice on a high-AGEs diet developed ectopic calcification of the CEP and hypertrophy of NP cells (increased expression of COL-X) [104]. In cadaveric specimens, ectopic calcification of the CEP was found in IVDs at different stages of degeneration and was colocalized with methylglyoxalhydroimidazolone-1- (MG-H1-) positive cells. MG-H1 also colocalized with collagen 10 (COL10) and osteopontin (OPN) [105]. This implicates AGEs in CEP calcification and the osteogenic differentiation of NP cells. Similar results were found in bovine tail and cadaveric NP cells, and the AGEs/RAGE axis may be involved in AGEs-induced hypertrophy and osteogenic differentiation of NP cells [105]. Therefore, RAGE has potential as a therapeutic target.

CEP is the main channel for material exchange between the IVD and the body. CEP calcification results in cell loss and lacunar occlusion, restricting the diffusion of nutrients and metabolic waste [14]. This leads to a decrease in glucose concentration and an increase in metabolic waste in the IVD [14], inducing degenerative changes. CEP sclerosis restricts the entry of drugs and biological agents into the IVD from the blood, influencing IVD-related diseases. AGEs promote CEP thickening and calcification [93]. Reducing AGEs deposition in the IVD and blocking the related signal pathway could ameliorate CEP calcification and reduce the level of metabolic waste in the IVD. In an *in vitro* experiment, MMP-8 treatment of the cadaveric lumbar CEP reduced the sulfated GAG and local collagen levels and altered collagen structure, thereby improving the diffusion of a small solute (376 Da). Also, the effect of MMP-8 was negatively correlated with AGEs content [106]. There is a marked difference between the *in vitro* cadaver experiment and the *in vivo* environment. Cellular electrical activities and the osmotic pressure between tissues need to be considered.

### 3.5. Inflammation

A painful IDD is in a chronic inflammatory state, and proinflammatory cytokines are upregulated in symptomatic IDD [107–109]. Age, smoking, infection, biomechanical abnormalities, and malnutrition can result in abnormal IVD cells and the production of cytokines and catabolic factors [17, 109–112]. Although the importance of these factors is unclear, they decrease the water signal of the IVD on T2-weighted MRI, known as *black disc*, as well as inflammation and NP herniation [109]. However, AGEs accumulation is also associated with increased inflammation [113]. AGEs accumulation in the IVD leads to increased expression of the proinflammatory factor TNF-*α* [92], which is associated with disc herniation and nerve stimulation and ingrowth [114, 115]. Intradiscal injection of AGEs in mice increased IL-23 expression and decreased the level of the anti-inflammatory cytokine IL-10 [84]. This implicates AGEs in the development of intradiscal inflammation. High mobility group box 1 (HMGB1) and IL-1*β* regulate the release of inflammatory factors from the degenerated IVD via RAGE. Because it is a multiligand receptor [116], it is uncertain whether RAGE is associated with AGEs. Cytokines are important players in IDD [109], and further work should focus on their regulation by AGEs in IDD.

### 3.6. Discogenic and Radicular Pain

Discogenic low back pain and radicular pain caused by IDD are common musculoskeletal diseases of unknown pathogenesis with no effective treatment. The serum methylglyoxal (MG) level in patients with lumbar disc herniation (LDH) with pain is higher than in painless or normal volunteers and is correlated with the visual analog scale score [117]. In an NP implantation animal model, simulated lumbar disc herniation increased the MG level in serum and the dorsal root ganglion (DRG), leading to mechanical pain and increased DRG neuron activity. This is accompanied by a decrease in the activity of glyoxalase 1 (catalyzes MG hydrolysis). The MG scavenger aminoguanidine can reduce MG accumulation in the DRG and ameliorate the mechanical pain caused by NP implantation and enhanced DRG neuron activity [117]. Also, activation of the RAGE/STAT3 pathway is key in LDH-induced persistent pain and so may be a therapeutic target [118]. Diabetic mice showed prolonged radicular pain-related behaviors, likely to be associated with prolonged inflammation and nerve regeneration under diabetic conditions [118].

AGEs accumulation destroys the normal structure of the IVD [92], leading to diseases such as intervertebral disc herniation. In disc herniation, CD68^+^ macrophages, neutrophils, and T cells (CD4^+^ and CD8^+^) migrate into the disc due to the ingrowth of blood vessels [119, 120]; nerve fibers from the DRG also invade IVD tissue [121, 122]. The stimulation of nerve fibers by components of the IVD may cause discogenic and/or radicular pain. Although AGEs destroy the integrity of the IVD structure, it may not only be the initiating factor in discogenic and radicular pain but also may be a potential therapeutic target in terms of its impact on the excitability of the DRG.

### 3.7. Intervertebral Disc Biomechanics

AGEs accumulation in the IVD gradually increases with age. A high-AGEs diet led to AGEs accumulation in the IVD, increasing IVD compressive stiffness, torque range, and torque to failure; these effects were more pronounced in females and were attributed to a marked increase in AGEs cross-linking in the AF [98]. AGEs accumulation also leads to collagen damage but does not affect its biomechanical properties and induces disc degeneration [99]. A high-fat diet can cause structural damage to the spine, and the degree of damage differs by gender [123]. Gender dependence is thus characteristic of the effects of several factors on spinal injury, but the mechanism is unclear.

Diabetes accelerates AGEs accumulation due to hyperglycemia [91]. In diabetic mice, AGEs accumulation resulted in a 97% increase in disc hardness [93]. In cadaver specimens, AGEs increased IVD hardness and AF mechanical stiffness, altering the biomechanical properties of the IVD [124]. AGEs reduce the water content of the AF and NP, which are related to the mechanical properties of the IVD, in a dose-dependent manner [125]. Although hyperglycemia in diabetes can lead to AGEs accumulation and destruction the of IVD, in the early stage of diabetes, especially in young patients, hyperglycemia may precede AGEs and cause changes in the structure of IVD. These changes may explain the development of IDD in patients with late-stage diabetes [126]. In general, with the increase of age, AGEs accumulate in the IVD, promoting collagen cross-linking and structural changes, decreasing metabolism, increasing catabolism, destroying the normal structure, and degrading the biomechanical properties of the IVD.

## 4. Conclusions and Perspectives

The integrity of the IVD is the necessary basis for its biomechanical properties. Any factor that causes structural changes will interfere with the normal function of the IVD. The accumulation of AGEs in IVD has caused extensive damage to various structures of IVD, including NP, AF, and CEP, resulting in the occurrence of IDD and becoming the basis of spinal degenerative diseases such as disc herniation and spinal stenosis. AGEs derived from endogenous and exogenous pathways (such as daily diet and smoking) can be deposited in IVD through the CEP and play a pathogenic role through the mitochondrial pathway, ER pathway, AGEs-RAGE axis, etc. The effect of high-AGEs diet on IVD may be gender dependent, and the effect on women is more obvious, but it is controversial. AGEs inhibitor treatment has a relatively clear effect on improving lesions. However, the route of administration must be considered. The operability of in vitro and in vivo tests is different, and oral administration may be more acceptable. However, CEP sclerosis may be unfavorable to the absorption of drugs and biological agents. There are few studies on improving permeability in patients with endplate sclerosis, and there is no effective treatment at present. There are many factors leading to IDD, and it is not clear which is relatively important at present. Existing studies show that AGEs have a wide impact on IVD and may be one of the important factors. Although we have a certain understanding of its pathogenic mechanism, there are still few studies on the whole, and some of them are controversial. There may be interference between different mechanisms. The underlying mechanism still needs to be further revealed to find potential molecular targets for effective treatment.

## Figures and Tables

**Table 1:** Classification of some AGEs based on chemical structure and ability to fluoresce.

AGE compound	Cross-linking	Fluorescence
Pyrraline	No	No
N-Carboxymethyllysine (CML)	No	No
N-Carboxyethyllysine (CEL)	No	No
Imidazolones	No	No
Glyoxal-lysine dimer (GOLD)	Yes	No
Methylglyoxal-lysine dimer (MOLD)	Yes	No
Pentosidine	Yes	Yes
Argpyrimidine	Yes	Yes
Vesperlysine A	Yes	Yes

## Data Availability

Contact the corresponding author to obtain relevant data.
